# Multimodal Exercise Effects in Older Adults Depend on Sleep, Movement Biography, and Habitual Physical Activity: A Randomized Controlled Trial

**DOI:** 10.3389/fnagi.2021.722799

**Published:** 2021-10-22

**Authors:** Oliver Vogel, Daniel Niederer, Lutz Vogt

**Affiliations:** Department of Sports Medicine and Exercise Physiology, Institute of Sports Sciences, Johann Wolfgang Goethe-University, Frankfurt, Germany

**Keywords:** sleep duration, cognitive performance, active aging, accelerometry, gait performance

## Abstract

**Background:** The promotion of healthy aging is one of the major challenges for healthcare systems in current times. The present study investigates the effects of a standardized physical activity intervention for older adults on cognitive capacity, self-reported health, fear of falls, balance, leg strength and gait under consideration of movement biography, sleep duration, and current activity behavior.

**Methods:** This single-blinded, randomized controlled trial included 49 community-dwelling older adults (36 women; 82.9 ± 4.5 years of age (Mean [M] ± SD); intervention group = 25; control group = 24). Movement biography, sleep duration, cognitive capacity, self-reported health status, and fear of falls were assessed by means of questionnaires. Leg strength, gait, and current activity levels were captured using a pressure plate, accelerometers, and conducting the functional-reach and chair-rising-test. The multicomponent intervention took place twice a week for 45 min and lasted 16 weeks. Sub-cohorts of different sleep duration were formed to distinguish between intervention effects and benefits of healthy sleep durations. Change scores were evaluated in univariate analyses of covariances (ANCOVAs) between groups and sub-cohorts of different sleep duration in both groups. Changes in cognitive capacity, self-reported health, fear of falls, balance, leg strength, and gait were investigated using the respective baseline values, movement biography, and current activity levels as covariates. Analysis was by intention-to-treat (ITT).

**Results:** We found sub-cohort differences in cognitive capacity change scores [*F*_(3,48)_ = 5.498, *p* = 0.003, η*p*^2^ = 0.287]. Effects on fear of falls [*F*_(1,48)_ = 12.961, *p* = 0.001, η*p*^2^ = 0.240] and balance change scores *F*_(1,48)_ = 4.521, *p* = 0.040, η*p*^2^ = (0.099) were modified by the level of current activity. Effects on gait cadence were modified by the movement biography [*F*_(1,48)_ = 4.545; *p* = 0.039, η*p*^2^ = 0.100].

**Conclusions:** Unlike for functional outcomes, our multicomponent intervention in combination with adequate sleep duration appears to provide combinable beneficial effects for cognitive capacity in older adults. Trainability of gait, fear of falls, and flexibility seems to be affected by movement biography and current physical activity levels.

**Trial registration:** This study was registered at the DRKS (German Clinical Trials Register) on November 11, 2020 with the corresponding trial number: DRKS00020472.

## Background

Following the demographic change and the herewith associated increase in civilization disease incidences, healthy aging has increasingly become a key responsibility of modern healthcare systems (Friedman et al., 2019). A sufficient amount and quality of sleep as well as current and lifetime physical activity are major contributors to healthy aging (Reid et al., 2006; Daskalopoulou et al., 2017; Gopinath et al., 2018).

Physical activity in the form of complying with guidelines, maintaining lifelong activity, and exercising is known to contribute to several indicators of health (Haskell et al., 2009; Piercy and Troiano, 2018; Ruegsegger and Booth, 2018). Health parameters affected by physical activity cover a wide variety, for instance, including an increase in functional capacity as well as the risk reduction of various diseases (Vlietstra et al., 2018; Posadzki et al., 2020). Health benefits associated with sleep behavior comprise, *inter alia*, cardiometabolic health, and adiposity (Chaput et al., 2020).

However, several sleep parameters as well as markers of habitual physical activity behavior are reported to deteriorate along with the aging process (Ohayon et al., 2004; Speakman and Westerterp, 2010). These age-related declines in physical activity and sleep quality have been associated with losses in cognitive and functional capacity (Gadie et al., 2017; Landi et al., 2018; Trieu and Alessi, 2019; Chaput et al., 2020). Consequently, the maintenance of physical activity levels and sleep quality contributes to healthy aging.

Besides general physical activity and sleep behavior, interventions integrating exercise (e.g., resistance or endurance training), motor-cognitive (e.g., dual-task walking, attention, and memory tasks), and mental [e.g., relaxation techniques or mindfulness based stress reduction (MBSR)] components promote healthy aging in older adults (Fessel et al., 2017; Jadczak et al., 2018). Such multicomponent interventions improved cognitive dimensions (executive function, memory, and working memory), health-related self-report measures (quality of life, fear of falling), and functional outcomes (postural control, gait, strength) (Cadore et al., 2013; Freiberger et al., 2013; Knobf et al., 2014; Tarazona-Santabalbina et al., 2016; Fessel et al., 2017; Northey et al., 2018). Further, dimensions of sleep like duration and efficiency were enhanced (Mendelson et al., 2016; Dolezal et al., 2017).

Besides direct effects on healthy aging, most health-related factors exhibit further indirect effects and mutual relationships. To date, evidence on the interaction between physical activity and sleep in humans is scarce. However, mutual effects evoked by growth hormones and signaling molecules [brain derived neurotrophic factor (BDNF) and interleukin-6 (IL-6)] are indicated (Tan et al., 2020). Consequently, beyond being an effector or outcome in intervention studies, sleep duration is related to physical activity (McClain et al., 2014; Mendelson et al., 2016). In addition, old age habitual physical activity is predicted by movement biography (Vogel et al., 2021a). According to that, sleep parameters and physical activities are not exclusively related to healthy aging, but exhibit mutual interactions and assumingly respond to multicomponent interventions. Sleep duration, current and lifetime physical activity may consequently modify exercise intervention effects. However, to our knowledge, no study evaluated their relation to, or impact on, exercise intervention effects. An increase in the effects and efficacy of multimodal exercise interventions might contribute to the promotion of healthy aging.

The present study investigates the effects of a multicomponent exercise intervention for older adults stratified by sleep duration. We hypothesize that (1) the exercise intervention enhances cognitive and functional outcomes in comparison to controls and (2) effects differ by sleep duration.

## Methods

### Study Design

This single-blinded, randomized controlled trial was approved by a local review board (Goethe-University, Department of Psychology and Sports Sciences, Ethics Committee, Approval number: 2019-22) and conducted in accordance with the Declaration of Helsinki. All participants signed informed consent before inclusion.

### Participants

Inclusion criteria comprised a minimum age of 65 years, the capability of walking, and a community dwelling housing situation in a retirement home, combined with a self-reliant lifestyle. Exclusion criteria consist of dementia [Montreal cognitive assessment (MoCA)] <17 points (Freitas et al., 2018) and acute injuries or infections.

Recruitment was conducted at the respective residences of participants by personal contact subsequently to presentations of the study at meetings of residents. Measurements and intervention units were conducted at the respective residences of participants as well. The participants were recruited in three different institutions located in Frankfurt (Hessen, Germany) and surrounding areas.

The sample size was determined using G^*^Power (version 3.1). The calculation was based on cognitive capacity as primary outcome, assuming an α-error of 5%, β-error of 20%, and effect size of *F* = 0.235 (Papp et al., 2009; Mewborn et al., 2017; Gheysen et al., 2018). The sample size was determined for a fixed effects ANCOVA regarding main effects and interactions. The sample size calculation yielded a required total of 43 participants. Assuming a 20% dropout-rate, 52 participants were recruited (Chatfield et al., 2005).

### Experimental Setup

Block randomization was conducted to create two equal groups. Each participant was randomly assigned to either an intervention or control group (CG). Blinded allocation sequence compiling, participant enrollment, and participant assignment were performed by the director of studies. The allocation sequence for randomization was created using BiAS (BiAS for Windows, Frankfurt, Germany). Assessors were blinded to group allocation of the participants; the director of studies was not involved in the conduction of the intervention or assessments.

### Contents of the Multimodal Intervention

The intervention group (IG) received a multicomponent training, based on a previously published training protocol (Cordes et al., 2019). Frequency, intensity, and duration of the training exercises were adapted on a group level with regard to the assumingly higher performance in the investigated cohort of community-dwelling older adults. The intervention was conducted by a sports scientist, following a standardized manual. Training frequency was twice a week for 16 weeks, duration was 45 min. The exercise units were structured in (1) warm-up, (2) walking tasks, (3) sitting gymnastics, and (4) flexibility and relaxation exercises. The difficulty of exercises was increased over the 16 weeks by switching task complexity, increasing repetitions, or adding weights (Table 1). The progression of the difficulty of exercises was based on the perceived exertion of participants (Williams, 2017). Rating of perceived exertion (RPE) was conducted every 4 weeks to check if the participants are capable of the next increment of task complexity, intensity, and/or duration. An average RPE of <12 was used as an indicator to increase the level of difficulty.

**Table 1:** Intervention exercises.

**Category**	**Exercise**	**Intensity**				**Duration/repetitions**			
		**Week 1–4**	**Week 5–8**	**Week 9–12**	**Week 13–16**	**Week 1–4**	**Week 5–8**	**Week 9–12**	**Week 13–16**
Balance	Romberg-stance	X	-	-	-	30–60 s	-	-	-
	Semi-tandem stance	-	X	-	-	-	30–60 s	-	-
	Tandem stance	-	-	X		-	-	30–60 s	-
	Single leg stance	-	-	-	X	-	-	-	30–60 s
	Shifting weight between heels and toes	X	X	X	X	30–60 s	30–60 s	30–60 s	30–60 s
Coordination/ cognition	Throwing and catching chiffon cloths	Unilateral	Bilateral	Contralateral	Contralateral	3 min	3 min	3 min	3 min
	Throwing and passing balls	Both hands/One ball	One-handed/One ball	Both hands/One ball	One-handed/Multiple balls	4 min	4 min	4 min	4 min
	Single leg stance	No additional task	Drawing figures with the free leg	Drawing figures with the free leg	Drawing figures with the free leg	4 min	4 min	4 min	4 min
	Memory tasks	Variable complexity	Variable complexity	Variable complexity	Variable complexity	5 min	5 min	5 min	5 min
Gait/endurance	Walking at preferred speed	Normal step size/No additional obstacles	Large step size/No additional obstacles	Normal step size/Additional obstacles	Large step size/Additional obstacles	5–10 min	5–10 min	5–10 min	5–10 min
	Fast walking	Normal step size/No additional obstacles	Large step size/No additional obstacles	Normal step size/Additional obstacles	Large step size/Additional obstacles	5–10 min	5–10 min	5–10 min	5–10 min
	Intermitted walking	Sitting and standing up task every 20 m	Sitting and standing up task every 20 m	Sitting and standing up task every 10 m	Sitting and standing up task every 10 m	3–5 min	3–5 min	3–5 min	3–5 min
	Intermitted walking	Stopping and starting at signal	Stopping and starting at signal	Stopping and starting at signal	Stopping and starting at signal	3–5 min	3–5 min	3–5 min	3–5 min
	Dual task walking	-	Recognizing or reacting to signs	Recognizing or reacting to signs	Recognizing or reacting to signs	-	3–5 min	3–5 min	3–5 min
Strengthening	Knee extension and flexion (sitting)	Bodyweight	1 kg weight bands	2 kg weight bands	2 kg weight bands	3 × 15–20 repetitions	2 × 15–20 repetitions	1 × 15-20 repetitions	2 × 15–20 repetitions
	Upright rowing	Empty bar	Empty bar	Empty bar	Empty bar	1 x 15 repetitions	1 x 15 repetitions	2 x 15 repetitions	2 x 15 repetitions
	Upper body rotation	X	X	X	X	1 x 10 repetitions to each side	1 x 10 repetitions to each side	2 x 10 repetitions to each side	2 x 10 repetitions to each side
	Compressing and uncompressing the bar held horizontal in front of the body	Subjective exhaustion	Subjective exhaustion	Subjective exhaustion	Subjective exhaustion	30 s each direction	30 s each direction	60 s each direction	60 s each direction
	Compressing a towel with knees/ arms	X	X	X	X	10 x 3 s	15 x 3 s	10 x 5 s	15 x 5 s
	Pulling on a towel held in front of the body	X	X	X	X	10 x 3 s	15 x 3 s	10 x 5 s	15 x 5 s
	Lifting Legs (Sitting)	Variable lifting height	Variable lifting height	Variable lifting height	Variable lifting height	4 min	4 min	4 min	4 min
	Clenching fists and tensing arm muscles	X	X	X	X	15 x 5 s	15 x 5 s	15 x 5 s	15 x 5 s
	Standing up (no arms)	X	X	X	X	10 repetitions	15 repetitions	20 repetitions	20 repetitions
	Biceps curls	2 kg	3 kg	4 kg	5 kg	3 x 10 repetitions	3 x 10 repetitions	3 x 10 repetitions	3 x 10 repetitions
	Reaching above head	1 kg	1 kg	2 kg	2 kg	3 x 10 repetitions	3 x 10 repetitions	3 x 10 repetitions	3 x 10 repetitions
	Front raises (straight arm)	Bodyweight	Bodyweight	1 kg	1 kg	3 x 10 repetitions	3x 10 repetitions	3 x 10 repetitions	3 x 10 repetitions
Stretching/ Cool-down	Progressive muscle-relaxation	X	X	X	X	5–10 min	5–10 min	5–10 min	5–10 min
	Porcupine ball self massage	X	X	X	X	5–10 min	5–10 min	5–10 min	5–10 min
	Imaginary journey	X	X	X	X	5–10 min	5–10 min	5–10 min	5–10 min
	Stretching of the strained muscles	X	X	X	X	5–10 min	5–10 min	5–10 min	5–10 min

*X, Exercise was conducted; Exercise was not conducted; kg, Kilogramm*.

The warm-up and cool-down phase took 5 to 10 min each. The major part consisted of walking tasks and sitting gymnastics, lasting 15–20 mins each. Warm-up comprised movement games aiming to prepare the participants for the physical demands of the respective unit and relax the atmosphere by utilizing group-based games encouraging interaction. Walking tasks provided for endurance training, while simultaneous processing of dual tasks targeted cognitive capacity and balance. Walking distance was set to 15 m consistently, whereas, repetitions progressed from 15 to 25 walks per training session throughout the intervention period. Sitting gymnastics were utilized as the basis for strengthening exercises with intermittent coordination tasks serving as breaks in between physically strenuous exercises. Strengthening exercises progressed from 10 to 20 repetitions, from two to three sets and no additional weight to 1 or 2 kg. Balancing tasks progressed in the form of switching body position (e.g., sitting, standing, standing on one leg) to a higher level of difficulty throughout the intervention period. Cool-down comprised stretching for flexibility and ending the exhaustive exercises. The relaxation part was supposed to increase physical and mental well-being. The intervention was instructed as group course of maximum 10 participants.

The waiting-list CG received no additional training and was told to keep up their habitual activity levels during the intervention period.

### Effect Estimator Outcomes

The effect estimator outcomes were assessed by sports scientists prior to and immediately following the 16-week intervention. The intervention and measurements were conducted by different sports scientists to assure blinding. The outcomes comprised cognitive capacity, subjective health status, fear of falling, leg strength, balance, and gait parameters.

Cognitive capacity, as the primary outcome, was captured by means of MoCA; a 12-item screening tool for executive function, visuospatial abilities, language, attention, concentration and working memory, abstract reasoning, memory, and orientation. On a scale of 0 to 30 points, a higher value represents a better cognitive capacity. We utilized the total score for further processing. The MoCA shows satisfactory psychometric properties in older adults (Thomann et al., 2020).

Self-reported health status was captured by means of the Shortform-12 (SF-12), a reduced version of the SF-36. Asking for physical and mental health in 12 items, the questionnaire shows adequate psychometric properties (Drixler et al., 2020). The SF-12 yields a mental and physical score we utilized for further processing. Higher scores (mental, physical) represent better self-rated health.

Falls self-efficacy was captured by means of the German version of the abbreviated Falls Efficacy Scale (Short FES-I). The version was reduced from 16 to 7 items for concerns of falling in different situations. A higher total score value represents more concerns of falling. We utilized the total score for further analyses. Psychometric properties of the short version reach satisfactory levels in older adults (Kempen et al., 2007, 2008). The outcome of the FES-I questionnaire is often referred to as “fear of falling” in the literature. In the following, we adopt the wording of “fear of falling,” under consideration of the original wording of the outcome (fall-related self-efficacy). Despite its interchangeable use in the literature and its relation, we explicitly state that fear of falls and falls self-efficacy are not the same. All questionnaires were completed by, or with the help of, assessors in form of interviews.

For leg strength rating, assessed by the chair-rising-test, the participants were asked to stand up from a chair five times in a row with their arms crossed in front of their chest. The time required to complete the task was measured by the assessor using a standard stopwatch. A shorter time to complete the test indicates higher levels of leg strength. The chair-rising-test exhibits excellent reliability values and is confirmed as a safe test protocol in older adults (Melo et al., 2019; Mehmet et al., 2020).

The functional-reach is a recommended motor assessment, exhibiting adequate psychometric properties (Trautwein et al., 2019; Arora et al., 2020). We conducted the sitting version of the test to minimize the risk of falls in our cohort of older adults. Hence, the starting position was sitting on a chair in a neutral body position, arms stretched to the front. From the starting position, the participants were asked to reach forward as far as possible without losing contact with the sitting surface of the chair. The distance between starting and maximum reach position was measured by means of a commercial measuring tape. A further reach indicates higher levels of balance.

Spatiotemporal gait parameters (step-length, cadence, speed, double support phase, and walk-ratio) were recorded by a capacitive force-measuring platform (WinFDM v0.0.411, Zebris GmbH, Isny, Germany). Step-length is measured as the distance from toe to contralateral toe on a centered line describing the direction of movement. Cadence was calculated as steps per minute, gait speed was reported as distance (meters) walked per time (seconds). Double support phase described the percentage duration of a gait cycle with both feet touching the ground. The walk-ratio is calculated as the quotient of step-length and cadence. Adequate validity has recently been proved for the utilized platform (Rudisch et al., 2021). Participants were asked to walk at a comfortable, self-chosen speed along a 6-meter ground-level walkway containing the measuring platform. For valid results, participants crossed the walkway three times.

### Effect Modifiers

The potential effect modifiers were gathered prior to the commencement of the intervention period. Movement biography, sleep duration, and current activity levels were assessed.

Movement biography was captured by means of the Lifetime Leisure Physical Activity Questionnaire (LLPAQ). The LLPAQ captures lifespan physical activity from birth up to 95 years of age. The questionnaire records time spent in different activities over lifetime divided into seven periods. The questionnaire distinguishes between leisure and household activities. Furthermore, it asks for occupational activities and locomotion. For further processing, the sum of leisure activity induced energy metabolism [Metabolic Equivalent of Task-hours (METH)] from age 20–80 years was calculated. Adequate validity and good reliability have already been proved in a cohort of older adults (Vogel et al., 2020).

Sleep parameters were queried by means of the Pittsburgh Sleep Quality Index (PSQI), which exhibits acceptable psychometric properties in oldest-old populations (Zhang et al., 2020). The questionnaire gathers data on seven components contributing to: (1) subjective sleep quality, (2) sleep latency, (3) sleep duration, (4) sleep efficiency, (5) sleep disturbances, (6) sleeping-pill intake, and (7) daytime sleepiness (Buysse et al., 1989). For further processing, raw data on sleep duration were utilized (hours).

Both questionnaires were completed as an interview.

Current activity levels were recorded by uniaxial accelerometers (MyWellness Key, Technogym, Gambettola, IT) worn near the hip for 7 consecutive days. Datasets of at least four valid days (wear time of at least 10 h) were considered valid (Gabrys et al., 2015). Subject to measurement was physical activity [measured as energy expended for acceleration of the body (METH)] in total and in different intensity ranges, predefined by the accelerometers (1.8–2.9 MET; 3–5.9 MET; >6 MET). The accelerometers exhibit sufficient measurement properties in cohorts of older adults *via* cross-validation (Colbert et al., 2011; Gardiner et al., 2011; Herrmann et al., 2011; Bergamin et al., 2012; Sieverdes et al., 2013).

### Data Processing

Each group was further sub-stratified using cutoffs for sleep duration. Here, a subgroup who reported sleeping in recommended extents (REs) and one reporting divergent extents (DEs) was stratified in each, the IG and CG. An “optimal” sleep duration appears to depend on age and activity level of the investigated cohort as well as on the investigated outcome. Hence, we conducted a preliminary study and screened the literature for appropriate studies to determine tailored cutoffs. REs were defined as 5–7 h of sleep, as this period is found to be an intersection of results and findings of our preliminary study regarding health-beneficial sleep durations in the literature (Ferrara and de Gennaro, 2001; Gangwisch et al., 2008; Bellavia et al., 2014; Hall et al., 2015; Devore et al., 2016; Silva et al., 2016; Vogel et al., 2021b). Consequently, DEs concern any sleep duration of <5 or more than 7 h.

The LLPAQ data were translated to METH and overall activity from the age span 20–80 years was summed. Activity levels below the age of 20 were not taken into account due to an expectedly high reporting bias (Vogel et al., 2020).

The change scores of primary and secondary outcomes were computed by subtracting initial measurement values from follow-up measurement values. According to a *√v*-dependency shown in literature, step-length, and cadence (captured in gait analysis) were normalized to speed for further processing (Winter, 2009).

### Statistics

Statistical programs used were statistical package for social sciences (SPSS) for Windows (Version 22, IBM, SPSS Inc., Chicago, IL, USA) and biometrical analyses of samples (BiAS) for Windows (Version 9.05, Goethe-University Frankfurt, Germany). A *p*-value of 5% was considered as a relevant cutoff for all significance testing.

Normal distribution (Shapiro-Wilk test; *p* > 0.05) and homoscedasticity (Levene test; *p* > 0.05) were both checked and given.

Baseline sub-cohort differences were analyzed by means of univariate ANOVA. Differences between genders (male vs. female), body mass index (BMI)–(18.5–24.9 vs. <18.5 & >24.9), and mild cognitive impairment (MCI)-ratings (<23 MoCA points vs. ≥23 MoCA points) were analyzed using the *t*-tests.

Intervention effects were evaluated using univariate ANCOVAs (4 × 1 ANCOVA) for each outcome in an intention-to-treat (ITT) analysis. Missing values were complemented by mean substitution. Randomized groups and sub-cohorts of different sleep duration were used for grouping (independent variable), and change scores of the effect estimating variables as the dependent variables. The potential effect modifiers (baseline values, habitual physical activity, and movement biography) were used as covariates.

*post-hoc* analyses used Fisher's Least Significant Difference (LSD) test to explore differences between individual groups.

## Results

**Figure 1:**
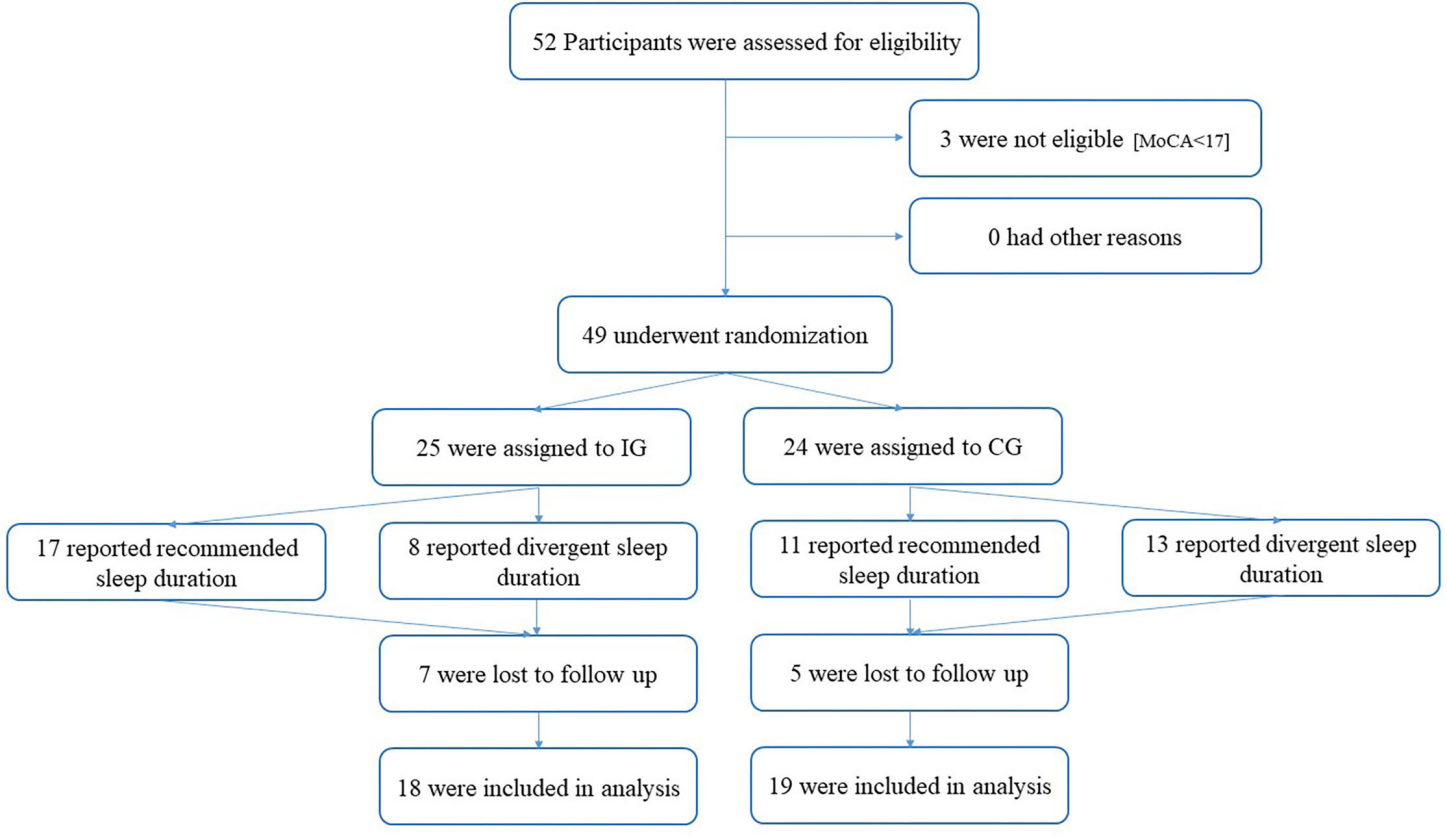
Participant flow. IG, Intervention Group; CG, Control Group; MoCA, Montreal Cognitive Assessment).

Out of the 52 recruited participants, three participants were excluded for not meeting the inclusion criteria of a MoCA threshold of 17 points. Of the remaining 49 [36 women; 82.9 ± 4.5 years of age (M ± SD)] randomly assigned participants, 25 received treatment. Overall dropout from both groups comprised 12 participants (7 IG; 5 CG). Reported reasons for dropout were a lack of interest or motivation, and health status of spouses. No adverse events occurred. There was no significant difference regarding baseline measures between participants that dropped out or stuck to the intervention. Participants flow is given in Figure 1.

Baseline demographic characteristics are charted in Table 2.

**Table 2:** Baseline demographic characteristics.

	**Intervention group-recommended extents**		**Intervention group-divergent extents**		**Control group-recommended extents**		**Control group-divergent extents**		**Group differences**	**Total**	
N	17		8		11		13			49	
Female	11		7		8		10		*F* = 0.563; *p* = 0.645	36	
	**Mean**	**SD**	**Mean**	**SD**	**Mean**	**SD**	**Mean**	**SD**		**Mean**	**SD**
Age [years]	83.4	4.4	83.5	4.9	82.2	4.8	82.4	4.8	*F*_(3,48)_ = 0.219; *p =* 0.882	82.9	4.6
Height [cm]	168.8	8.2	163.9	8.2	169.1	10.5	167.5	8.2	*F*_(3,48)_ = 0.689; *p =* 0.568	167.7	8.7
Weight [Kg]	66.0	9.9	56.1	9.8	66.4	13.4	72.2	12.7	*F*_(3,48)_ = 3.325; *p =* 0.039	66.0	12.3
BMI	23.1	2.6	20.9	3.2	23.1	2.9	25.5	3.1	*F*_(3,48)_ = 3.402; *p =* 0.037	23.3	3.2
MoCA [POINTS]	25.2	3.1	24.9	2.9	25.0	2.7	24.2	2.9	*F*_(3,48)_ = 0.314; *p =* 0.082	24.8	2.9
Current activity [moves/day]	501.7	259.9	357.8	202.7	411.7	279.6	268.8	158.8	*F*_(3,48)_ = 1.150; *p =* 0.424	369.9	233.7
Lifetime-activity [Total METh]	189,371	176,874	127,556	115,693	170,187	163,531	301,163	276,809	*F*_(3,48)_ = 0.461; *p =* 0.717	199,707	188,559

*CM, Centimeters; KG, Kilogramm; MOCA, Montreal Cognitive Assessment; METH, Metabolic Equivalent-Hours; SD, Standard Deviation*.

The sub-cohorts differed regarding body weight and BMI at baseline [body weight: *F*_(3,48)_ = 3.325; *p* = 0.039; BMI: *F*_(3,48)_ = 3.402; *p* = 0.037]. Baseline measurement of self-report health status yielded a physical score of (mean) 42.9 ± (SD) 11.0 points and a mental score of 53.9 ± 8.3 points. The baseline cognitive capacity was 24.8 ± 2.9 points (MoCA) and fear of falls 9.9 ± 3.5 points (FES-I). Balance and leg strength testing resulted in 36.0 ± 9.5 cm at “functional-reach” and 15.7 ± 5.3 s in the “Chair-Rising” -test. Gait analyses revealed a step-length of 55.9 ±7.3 cm and cadence of 110.0 ± 17.2 steps/min, both normalized to individual gait speed. Average walking speed was 1.0 ± 0.2 m/s, double support phase was 34.1 ± 7.6%, and the walk-ratio was 5.3 ± 1.3 mm/(step/min). Baseline and postintervention sub-cohort values for each measurement are shown in Table 3.

**Table 3:** Effect estimator values at baseline and post-intervention for each sub-cohort.

	**Intervention group-recommended extents**		**Intervention group-divergent extents**		**Control group-recommended extents**		**Control group-divergent extents**	
**Time of measurement**	**Pre**	**Post**	**Pre**	**Post**	**Pre**	**Post**	**Pre**	**Post**
Montreal Cognitive Assessment [points]	25.2 ± 3.1	26.4 ± 3.1	24.9 ± 2.9	23.6 ± 4.2	25.0 ± 2.7	24.7 ± 2.8	24.2 ± 2.9	21.0 ± 3.9
Falls efficacy scale international [points]	9.7 ± 2.5	9.5 ±1.9	10.0 ± 3.5	9.4 ± 2.5	9.7 ± 3.7	9.3 ± 3.8	10.4 ± 4.8	10.9 ± 4.9
Shortform 12 mental score [points]	54.5 ± 7.9	53.9 ± 8.2	52.9 ± 9.8	54.6 ± 11.0	57.7 ± 4.8	55.1 ± 7.9	51.6 ± 10.0	53.0 ± 11.3
Shortform 12 physical score [points]^***^	46.3 ± 10.7	46.7 ± 10.7	43.3 ± 9.6	42.3 ± 15.3	45.3 ± 10.1	48.8 ± 12.4	35.2 ± 10.3	36.3 ± 10.7
Funtional reach test [cm]	37.5 ± 8.5	38.5 ± 7.7	33.2 ± 12.6	36.6 ± 8.9	38.4 ± 6.7	36.4 ± 5.3	33.2 ± 10.5	33.8 ± 13.2
Chair-rise test [seconds]	15.8 ± 7.5	15.6 ± 8.1	16.8 ± 6.5	14.4 ± 5.3	15.0 ± 3.6	12.0 ± 1.7	15.0 ± 4.9	12.9 ± 4.0
Gait–speed [m/s]	1.03 ± 0.21	1.12 ± 0.12	0.93 ± 0.30	1.07 ± 0.21	1.01 ± 0.26	0.98 ± 0.33	0.94 ± 0.23	0.98 ± 0.27
Gait–cadence [steps/min]	103 ± 8	103 ± 7	110 ± 18	108 ± 12	107 ± 11	107 ± 17	122 ± 27	125 ± 25
Gait–step-length [cm]	58.7 ± 4.4	58.6 ± 4.2	55.8 ± 8.8	55.9 ± 5.5	56.5 ± 6.0	57.2 ± 7.8	51.1 ± 10.1	49.4 ± 8.9
Gait–double support phase [% of gait cycle]	32.5 ± 4.2	32.1 ± 4.1	34.6 ± 7.0	34.2 ± 7.7	31.9 ± 7.1	32.4 ± 5.7	39.5 ± 10.7	38.2 ± 10.4

*** *Sub-cohort differences at baseline measurement (p < 0.05)*.

*CM, Centimeters; M/S, Meters per second; Steps/Min, Steps per Minute*.

**Figure 2:**
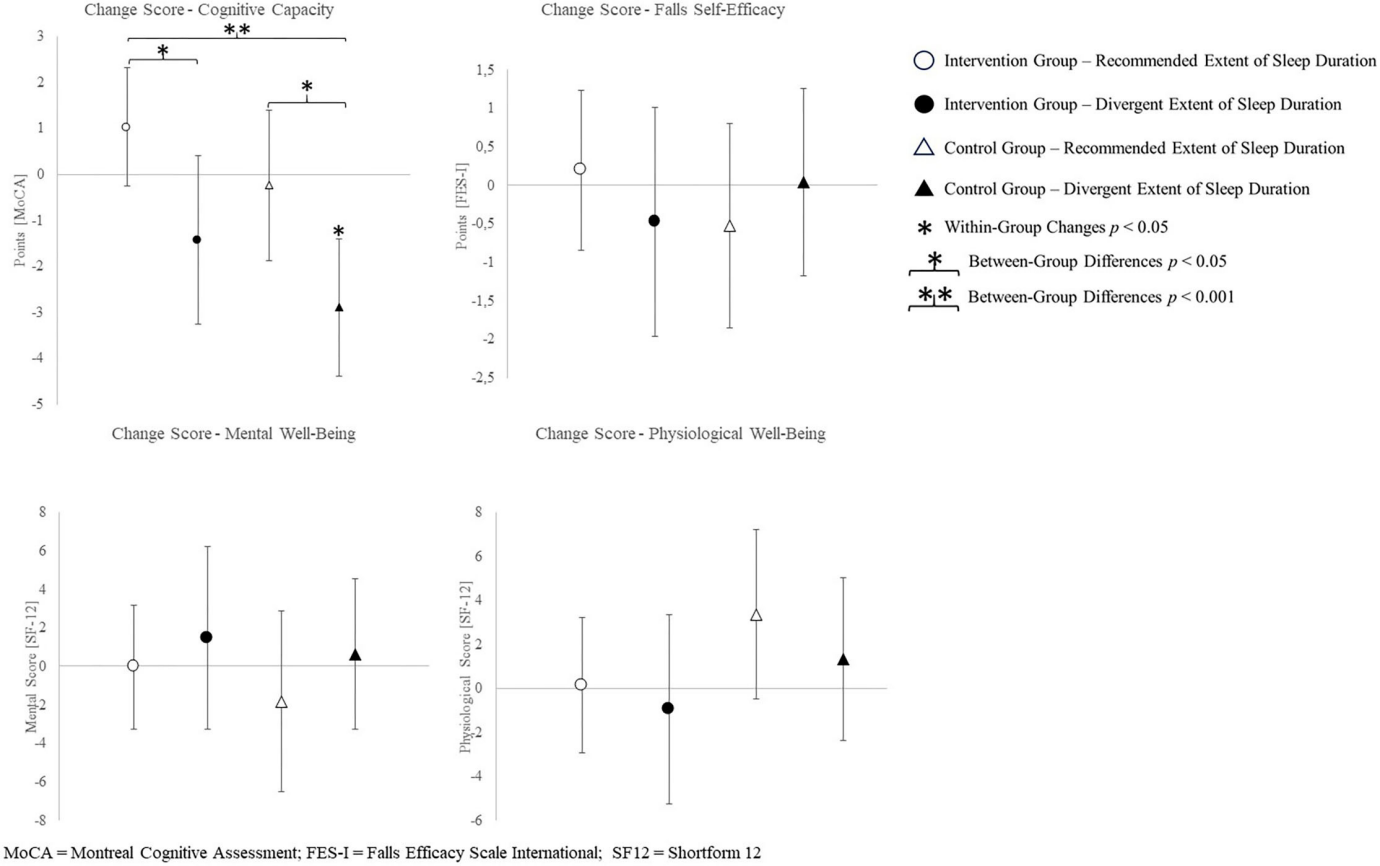
Change scores of the primary outcome cognitive capacity, measured by the Montreal cognitive assessment and secondary outcomes fear of falling and health-related quality of life.

**Figure 3:**
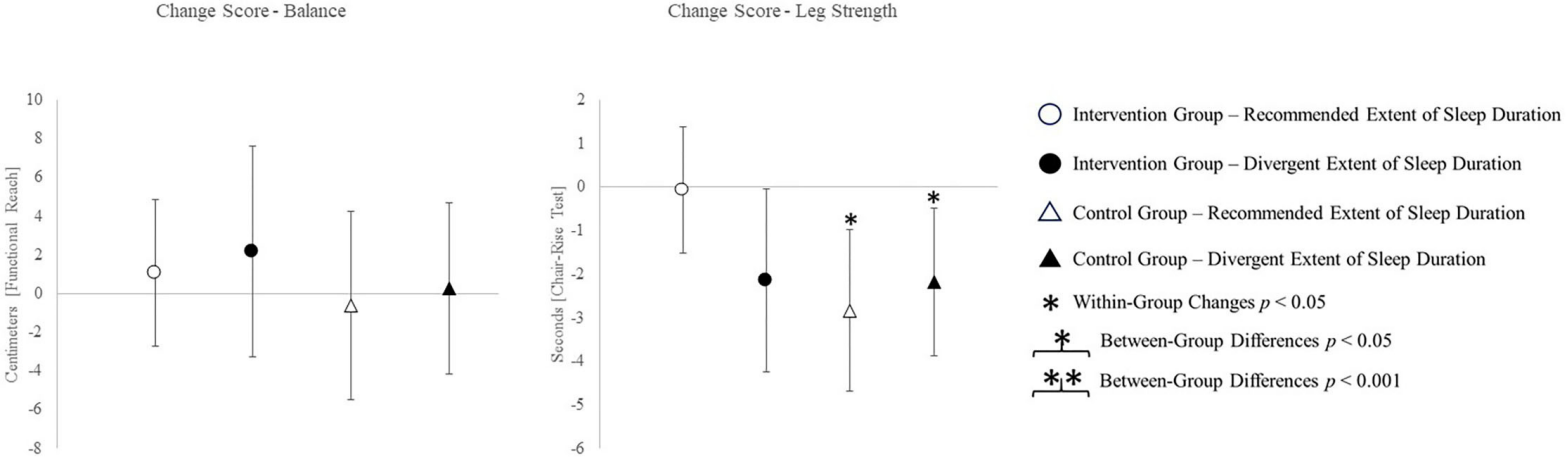
Change scores of secondary outcomes measured by functional-reach and chair-rise test.

**Figure 4:**
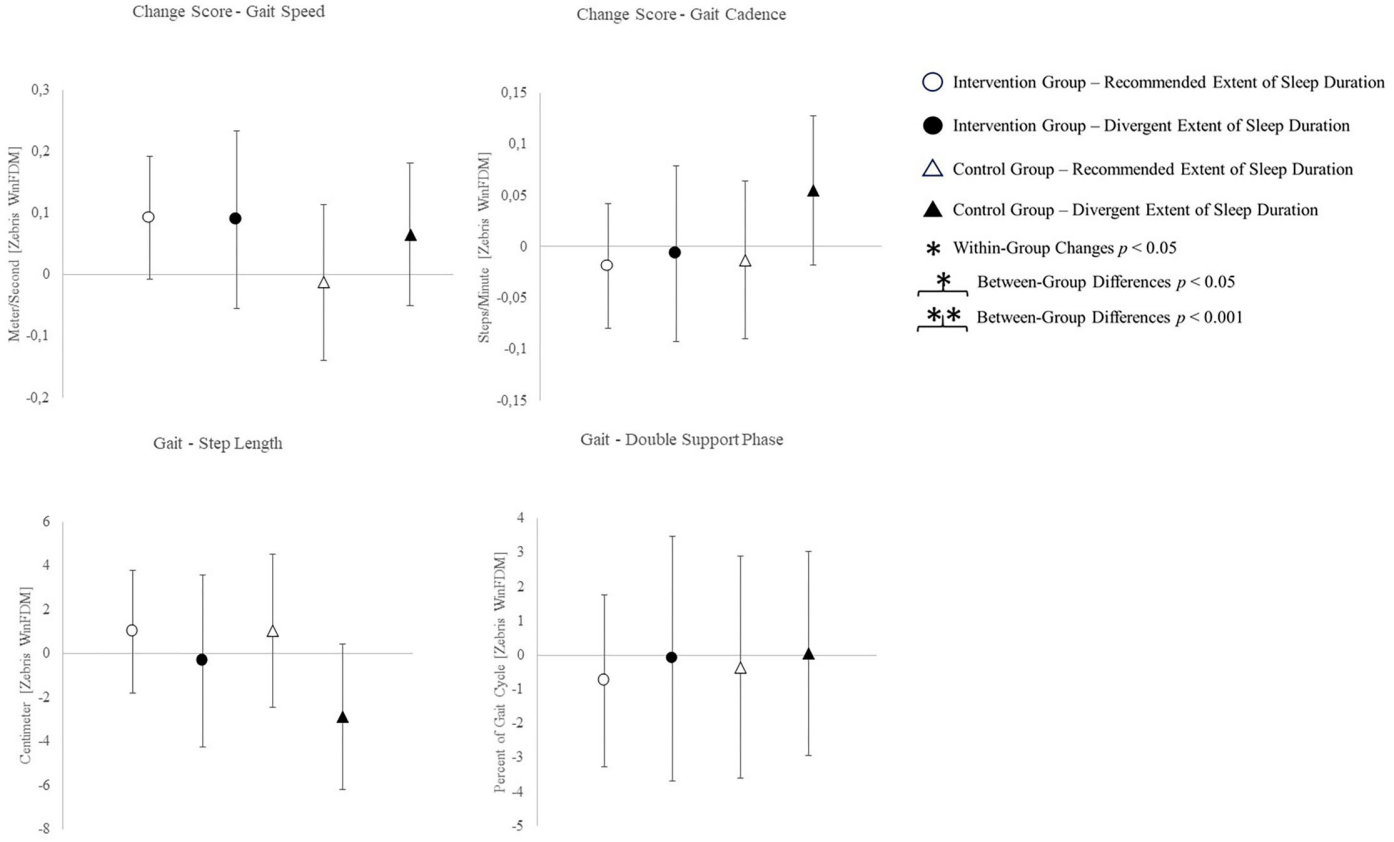
Change scores of the outcomes of gait analyses. Means and CIs are displayed.

The change scores of effect estimators are shown in the Figures 2–4. Significant differences between groups were found for change scores of cognitive capacities [*F*_(3,48)_ = 5.498, *p* = 0.003, η*p*^2^ = 0.287]. Balance, leg strength, and gait parameters showed no sub-cohort differences (each *p* > 0.05). Changes in fear of falls (FES-I) and balance change scores differed between groups, a contribution of the covariate “current activity levels (accelerometers)” was given [fear of falls: *F*_(1,48)_ = 12.961, *p* = 0.001, η*p*^2^ = 0.240; functional reach: *F*_(1,48)_ = 4.521, *p* = 0.040, η*p*^2^ = 0.099]. Change scores of gait cadence differed between groups (movement biography as the significant covariate: *F*_(1,48)_ = 4.545; *p* = 0.039, η*p*^2^ = 0.100). No other between-group-differences in any gait characteristic occurred (*p* > 0.05) (Table 4).

**Table 4:** ANCOVA results regarding intervention effects for the independent variable (group allocation) and covariates (baseline values, movement biography, and current activity).

	**Group allocation**			**Baseline values**			**Movement biography**			**Current activity**			**Estimated marginal means**			
	***F***	***p***	*** $\eta_{p}^{2}$ ***	***F***	***p***	*** $\eta_{p}^{2}$ ***	***F***	***p***	*** $\eta_{p}^{2}$ ***	***F***	***p***	*** ${\text{η}}_{p}^{2}$ ***	***M***	***SE***	***UL***	***LL***
Montreal cognitive assessment	5.498	0.003	0.287	4.320	0.044	0.095	3.376	0.073	0.076	2.131	0.152	0.049	−0.884	0.383	−1.657	−0.112
Falls efficacy scale international	0.357	0.785	0.025	15.780	<0.001	0.278	0.495	0.486	0.012	12.961	0.001	0.240	−0.189	0.312	−0.819	0.441
Shortform 12 mental score	0.403	0.752	0.029	2.160	0.149	0.050	0.622	0.435	0.015	2.000	0.165	0.047	0.051	0.994	−1.956	2.058
Shortform 12 physical score	0.910	0.445	0.062	0.065	0.801	0.002	0.178	0.675	0.004	0.948	0.336	0.023	0.957	0.901	−0.862	2.777
Funtional reach test	0.220	0.882	0.016	16.466	<0.001	0.287	0.272	0.605	0.007	4.521	0.040	0.099	0.710	1.136	−1.584	3.004
Chair–rise test	2.307	0.091	0.144	5.458	0.024	0.117	0.569	0.455	0.014	2.724	0.106	0.062	−1.813	0.437	−2.695	−0.930
Gait–speed	0.642	0.593	0.045	24.803	<0.001	0.377	3.747	0.060	0.084	3.162	0.083	0.072	0.058	0.030	−0.002	0.119
Gait–cadence	0.865	0.467	0.060	11.706	<0.001	0.222	4.545	0.039	0.100	0.149	0.702	0.004	0.004	0.018	−0.032	0.040
Gait–step-length	1.221	0.314	0.082	18.463	<0.001	0.311	4.517	0.040	0.099	0.157	0.694	0.004	−0.298	0.819	−1.952	1.355
Gait–double support phase	0.062	0.980	0.005	9.413	0.004	0.187	0.955	0.334	0.023	0.495	0.485	0.012	−0.295	0.750	−1.809	1.219

*M, Mean; SE, Standard Error; UL, Upper Limit 95% Confidence Interval; LL, Lower Limit 95% Confidence Interval*.

The *post-hoc* results are (as an addition to the values) illustrated in Figures 2–4.

## Discussions

We found that, in older adults, a multimodal exercise intervention improved cognitive capacity in the IG sleeping at REs, but not in the IG exhibiting divergent sleep durations. In the CG exhibiting divergent sleep durations, the cognitive capacity decreased. Since there were no differences between particular intervention or sleep duration groups regarding functional outcomes, both hypotheses can only be verified for cognitive capacity.

### Sample Representativeness

Mean values above reference values were found for double support phase, mental health, functional-reach, and the chair-rising-test of the study cohort (+22.2% in comparison to 27.9% double support phase of the reference values; +44.0% in comparison to a summary score of 37.5 points of the reference values for the SF12 mental health score; +6.2% in comparison to the 33.9 cm seated functional reach of the reference values; +41.4% in comparison to 11.1 s of the reference values for the chair-rising-test) (Jakobsson, 2007; Thompson and Medley, 2007; Delbaere et al., 2010; Hollman et al., 2011; Bohannon et al., 2017; Carson et al., 2018; Freitas et al., 2018; Kawai et al., 2019; Rosa et al., 2019). Below-reference mean values were found for physical health (−18.7% in comparison to a summary score of 50.3 points of the reference values for the SF12 physical health score), gait speed (−20.6% in comparison to 1.26 m/s for the reference value), step-length (−8.8% in comparison to 61.3 cm step-length of the reference values), step cadence (−11.9% in comparison to 124.3 steps/min for the reference values), and walk-ratio [−11.7 in comparison to 6.0 mm/(step/min) for the reference values] (Hollman et al., 2011; Makizako et al., 2017; Bogen et al., 2018; Bergland and Strand, 2019; Kawai et al., 2019; Ramírez-Vélez et al., 2020). Conclusively, our sample is considered representative of the underlying population since the captured parameters are not consistently above or below average.

### Intervention Effects and the Role of Sleep Duration

We assume the BMI differences too low and BMI values too close to healthy values to affect outcomes and to refer to BMI as an indicator of unhealthy lifestyles in the present cohort. Furthermore, the sub-cohorts exhibited common BMI scores on average with no extraordinary values in individual participants (Peralta et al., 2018).

Cognitive capacity was improved by the intervention, in particular, in the sub-cohort who slept the recommended amount. In combination with exercise, medium sleep durations appear to beneficially affect cognitive capacity. Exceptional sleep durations, on the other hand, presumably have adverse effects (Lo et al., 2016). The latter is true, irrespective of the group allocation. Seniors with medium sleep duration are, thus, trainable by multimodal exercise interventions in view of a positive influence on cognitive performance. We also found an intervention effect on cognition in participants exhibiting excessively long or comparable short sleep durations. Yet, the isolated effect of recommended sleep durations on cognitive decline may be larger than the isolated effect of solely participating in the exercise intervention. Change scores of the groups exhibiting both or no factors indicate combinable effects of healthy sleep durations and exercise interventions.

The CG sub-cohort sleeping <5 h or more than 7 h exhibited the largest decline of the MoCA score in the 16-week study period. The MoCA scores of three participants from this sub-cohort showed baseline values above and postintervention values below a MCI cutoff score of 23 points (Thomann et al., 2020). Contrary, the IG sleeping at REs shows gains in cognitive capacity, contrasting the usual age-related decline. Two participants of this group exhibited baseline values below and postintervention values above the MCI cutoff (23 points) (Thomann et al., 2020). Both of these groups exhibit change scores above the minimum detectable change for the MoCA (Feeney et al., 2016). According to this, the combination of a healthy sleep duration and participation in an exercise intervention yields improvements regarding cognitive capacity in the present cohort.

Incongruent results regarding exercise induced increases in self-reported health scores have been reported (Dekker-van Weering et al., 2017). The present results align with findings on exercise interventions exclusively affecting extraordinary low levels of self-reported well-being (Moriyama et al., 2020). A predictive value of sleep complaints on self-reported health and an inverse association of sleep duration and physical health scores is indicated (Reid et al., 2006; Johnson et al., 2017). Sleep efficiency on the other hand shows a positive association with both, the physical and mental aspects of self-reported health (Reid et al., 2006). Therefore, consistent sleep values used for group allocation possibly account for low mental health changes. An intervention targeting changes in sleep quality, however, might lead to changes in sleep parameters and mental health likewise. Health-related quality of life physical sum score, the functional reach ability, and most gait characteristics showed no between-group or intervention effects. Exclusively, the CG sub-cohort sleeping <5 h or more than 7 h shows a reduction in step-length and increase in cadence. The walk-ratio, calculated from step-length and cadence (step-length/cadence=walk-ratio), has been reported to relate to the risk of falls in older adults (Nakakubo et al., 2018). Besides multicomponent interventions, certain sleep durations appear to affect the risk of falls as well (Fu et al., 2019). However, the subgroup neither participating in the intervention, nor sleeping at REs, exhibits an increased walk-ratio, which indicates a decreased risk of falls (walk-ratio change scores: IG-RE −0.369 ± 7.152; IG-DE −0.426 ± 11.807; CG-RE 1.830 ± 16.954; CG-DE −3.101 ± 14.600).

The missing relationship between concerns of falls and sleep parameters in the present study does not align with the findings of related studies. Divergent measurement methods might cause the lack of influence by sleep duration. Whereas, the present study measured the concerns of falling by questionnaire, related studies measured the risk of falling by means of objective measures of balance performance (Hita-Contreras et al., 2018; Serrano-Checa et al., 2020). Hence, subjective concerns and the objective risk of falling might differ regarding their relationship toward sleep parameters. Intervention efficiency on FES-I change scores might be owed to the cognitive status of participants. An even more detailed look into the MoCA data regarding the MCI cutoff criteria revealed a different profit in terms of FES-I scores in participants above and below the MCI cutoff (23 points) (Thomann et al., 2020). Participants with a lower risk of MCI (≥23 points) improved (lowered) their FES-I score throughout the intervention period, whereas participants presenting with a higher risk of MCI (<23 points) exhibited worsened (increased) FES-I scores.

Health-related quality of life—mental health showed no changes in the subgroup participating in the intervention and sleeping at REs, or in the CG sleeping excessively short or long. A tendential increase in the IG sleeping excessively short or long and a decrease in the CG sub-cohort who sleeps the recommended amount is indicated. This is somewhat surprising. Regarding the present data, the multicomponent intervention might enhance mental health, whereas sleeping at recommended durations in the present study might be related to adverse effects. Hence, the effects of the intervention and recommended sleep durations seem to cancel each other out in the present results, as well as divergent sleep durations and being allocated to the CG does. Related studies report poorer mental health outcomes in association with a reduction in sleep duration (Tang et al., 2017). As limits of sleep duration differ between studies (<6 h, 6–8 h, >8 h in related studies; <5 h and >7 h, 5–7 h in the present study), the “reduction” in sleep duration might be equivalent to the recommended sleep durations in the present study (Tang et al., 2017). Consequently, considering the variability of “optimal” sleep durations, the allegedly differing results might align nevertheless. Especially, regarding the outcomes on mental health, an active CG might have been useful. An active CG might have helped discriminating the effect of social interaction within the group and with the trainer from the effects evoked by the intervention itself.

Even more surprising was the improvement in the chair-rise performance in each group except the IG with the recommended amount of sleep. The sleep duration being most helpful for physical strength gains appears to differ from the range we defined as recommended. Related studies report lower physical performance in relation to excessively long sleep durations (Fu et al., 2017). However, the sleep durations utilized for grouping do not even overlap between the present and related study (7–9 h in the related study; 5–7 h in the present study). Therefore, inconclusive results regarding leg strength might be owed to divergent “optimal” sleep durations between different outcomes.

### Modifying Factors

Habitual physical activity and movement history were found to be relevant covariates when exercise effects in different sleep-duration cohorts are investigated. This potential interaction complies with the results of related studies (Newton, 2001; Seco et al., 2013; Pau et al., 2014; Dawe et al., 2018). In participants exhibiting high lifetime but low current activity levels, former benefits in balance skills might have already deteriorated (Rosa et al., 2019). The present cohort predominantly moved at low intensities (89.6% of the captured activity), complying with literature on activity of older adults (Troiano et al., 2008). The lack of moderate to vigorous physical activity (MVPA) prevents analyses of their isolated impact on the investigated outcomes, which might provide beneficial insights.

While most studies use a self-report measure for physical activity or objective measures for isolated MVPA, we objectively captured total amounts of physical activity. Therefore, subliminal activity not reported in questionnaires might confound findings on gait parameter relationships (Ciprandi et al., 2017; Hamacher et al., 2018).

### Limitations and Future Studies

To assess balance, we utilized the seated functional-reach test in favor of fall risk minimization. As the seated version is less demanding than the standing version, it might have caused a ceiling effect in balance performance. Even though the selection of exercises, intensity, and tempo was tailored to our cohort, individual and continuous customization of the exercise load possibly enhances intervention effects in cohorts of heterogenous fitness levels (Herold et al., 2019). Since a highly personalized training intensity does not reflect common practice, an individual approach might facilitate more definitive results at the expense of generalizability and practical applicability. Although sleep parameter improvements are rather evoked by moderate than high-intensity exercise, there might be a reverse relationship to other entities (Bullock et al., 2020). We recommend further studies to integrate complementing objective sleep measurements and individual, subjective reports on perceived intervention intensity for continuous adjustment. The number of participants dropping out exceeded the number of additionally recruited participants in our cohort. For longitudinal studies investigating older adults lasting 4 months or more, it might be advisable to recruit more than the recommended additional 20%. Furthermore, data on sustainability acquired from a follow-up measurement, as well as on autonomous continuation of comparable activities, might provide further insight regarding the effectiveness of interventions in terms of health promotion. An additional active CG might be useful for further research in terms of discriminating the effect of social interaction within the group and with the trainer from the effects evoked by the intervention itself.

### Generalizability and Practical Relevance

Since balance, leg strength, and health status of the present cohort corresponds to age-matched general population, we consider our results to be generalizable. As high degrees of personalization do not comply with usual group-based health promotion offerings, they are not suitable for increasing generalizability. Some imbalance between performance status and intervention-induced physical demands of individuals is not uncommon and favors generalizability (Uemura et al., 2013). Our findings on covariates appear to be generalizable as all measurements of utilized variables represent usual dimensions.

## Conclusions

We found health beneficial effects of a multicomponent intervention in consideration of habitual sleep duration, habitual physical activity, and lifetime activity. In particular, sleep duration and multimodal exercises affect cognitive capacity synergistically. Fear of falls and balance change scores depend on current physical activity levels, whereas gait cadence changes depend on lifetime physical activity. From a general perspective, multivariate analyses of intervention effects are warranted to systematically assess interactions as well as mediating and moderating effects of multiple health contributing factors within one analytic approach. Research on synergistic, moderating, and mediating effects of health factors possibly enables insight into their mechanisms of action (Ohrnberger et al., 2017; Whibley et al., 2019). Such findings might enable a deeper understanding of differing effectiveness between interventions and help to enhance existing interventions (Janssen et al., 2020). Furthermore, besides physical activity guidelines, promotion of sleep behavior recommendations in older adults might benefit the maintenance of a healthy lifestyle and in particular, contribute to the prevention of cognitive decline.

## Data Availability Statement

The datasets used and/or analysed during the current study are available from the corresponding author on reasonable request.

## Ethics Statement

The studies involving human participants were reviewed and approved by Goethe-University, Department of Psychology and Sports Sciences, Ethics Committee. The patients/participants provided their written informed consent to participate in this study.

## Author Contributions

LV, OV, and DN: study concept and design, analysis and interpretation of data, and preparation of manuscript. OV: acquisition of data. All authors have read and approved the manuscript.

## Conflict of Interest

The authors declare that the research was conducted in the absence of any commercial or financial relationships that could be construed as a potential conflict of interest.

## Publisher's Note

All claims expressed in this article are solely those of the authors and do not necessarily represent those of their affiliated organizations, or those of the publisher, the editors and the reviewers. Any product that may be evaluated in this article, or claim that may be made by its manufacturer, is not guaranteed or endorsed by the publisher.
